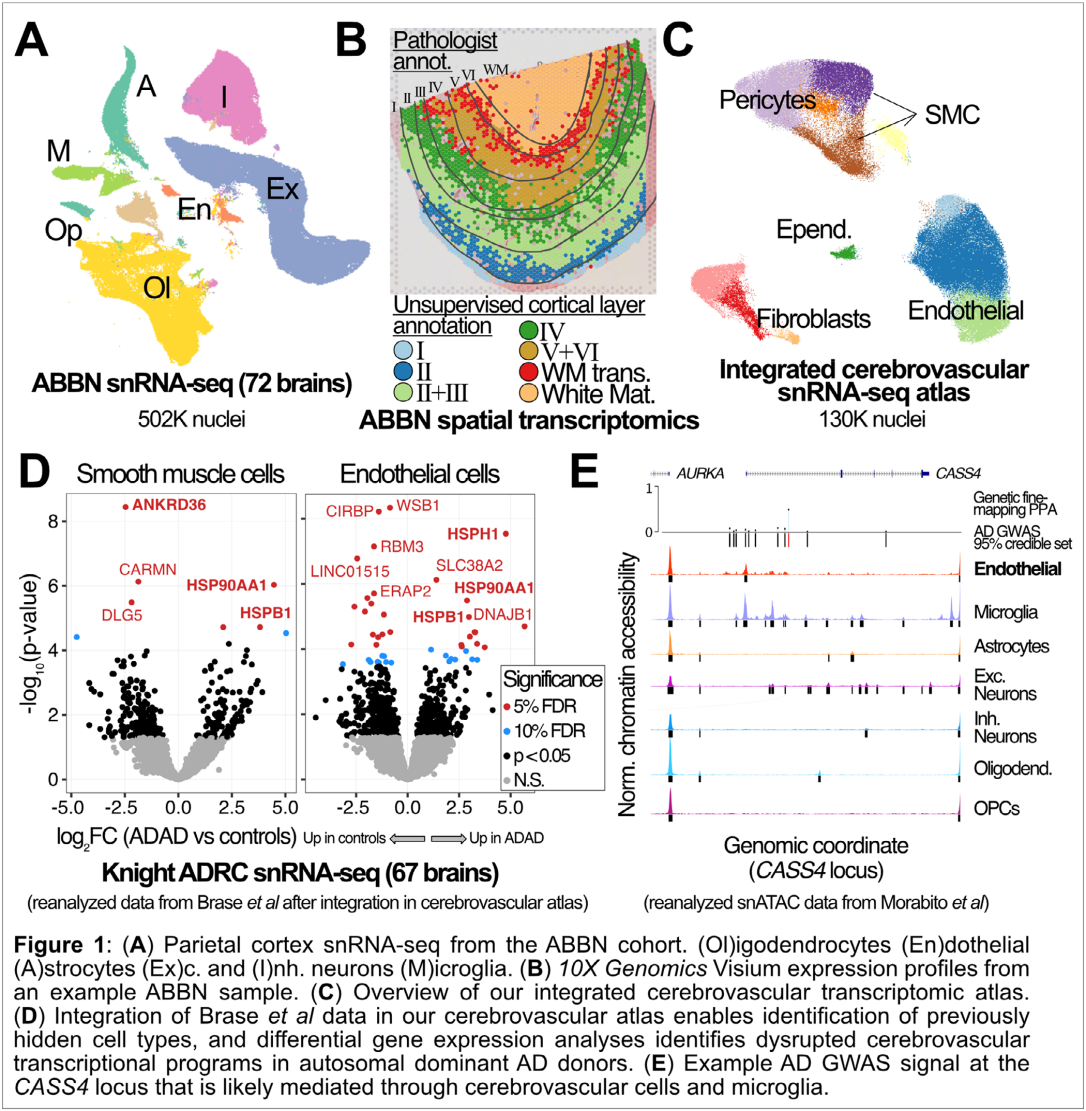# Uncovering the role of cerebrovascular phenotypes in AD through deep multi‐omic molecular profiling of human brains

**DOI:** 10.1002/alz.089238

**Published:** 2025-01-03

**Authors:** Ricardo D'Oliveira Albanus, Ekaterina Aladyeva, Taylor Bertucci, Logan Brase, Dominantly Inherited Alzheimer Network (DIAN), Bruno A. Benitez, Greg T Sutherland, Celeste M. Karch, Sally Temple, Oscar Harari

**Affiliations:** ^1^ Washington University in St. Louis, St. Louis, MO USA; ^2^ Neural Stem Cell Institute, Rensselaer, NY USA; ^3^ Beth Israel Deaconess Medical Center, Boston, MA USA; ^4^ University of Sydney, Camperdown, NSW Australia; ^5^ Ohio State University College of Medicine, Neurobiology of Aging & Resilience Center, Columbus, OH USA

## Abstract

**Background:**

The cerebrovasculature is an essential component of brain homeostasis. Cerebrovascular disorders are associated with an increased risk for neurodegenerative diseases, including Alzheimer’s disease (AD). However, the mechanisms by which cerebrovascular dysfunction contributes to neurodegeneration are poorly understood.

**Method:**

We optimized nuclei isolation from human brains to increase the representation of cerebrovascular cells (CVC) and performed single‐nucleus transcriptomic profiles (snRNA‐seq) of parietal cortex from healthy and AD donors from the Australian Brain Bank Network (ABBN; n = 72 brains), which are richly annotated for cerebrovascular phenotypes, including cerebral amyloid angiopathy (CAA). In parallel, we generated spatially resolved transcriptomic profiles for a subset of these samples. We integrated our new data with seven public snRNA‐seq datasets to create a comprehensive cerebrovascular atlas. Simultaneously, we reprocessed public snATAC‐seq data from cases and controls to identify regulatory elements in CVC likely mediating AD gene risk.

**Result:**

Our cerebrovascular atlas encompassed ∼133K CVC across five major cell types (endothelial, smooth muscle, pericytes, fibroblasts, ependymal). This high resolution allowed the identification of perturbed CVC transcriptional programs between cases and controls, including pronounced disruptions in cellular homeostasis via heat shock proteins and smooth‐muscle‐specific downregulation of *ANKRD36* in early‐onset AD patients. Rare variants in *ANKRD36* were recently implicated in AD in a whole‐exome sequencing study. We also identified transcriptional signatures associated with CAA in vascular and glial cells. We validated these results using spatial transcriptomics and data from the Seattle AD cohort. Lastly, we prioritized eight independent AD risk loci, including APP and APOE, where at least one fine‐mapped risk variant (95% credible set) overlapped a regulatory element active in vascular cells. At the *APH1B* locus, we identified a vascular enhancer likely regulating *TPM1* and *USP3*. All loci putatively acting through vascular cells were shared with at least one other cell type (*e.g., CASS4* with microglia), supporting reports of shared genetic risk between AD and cerebrovascular disease but suggesting the shared risk is mediated in a cell type‐specific manner (**Figure 1**).

**Conclusion:**

Our work provides novel insights into the role of cerebrovascular dysfunction in AD and identifies genes and regulatory elements mediating AD genetic risk through CVC.